# Evaluating Optical Coherence Tomography and X-Ray Computed Tomography to Measure Tablet Film Coat Thickness

**DOI:** 10.3390/pharmaceutics17091225

**Published:** 2025-09-20

**Authors:** Emily Sanchez, Trent Eastman, Jennifer Potter, Robert Meyer

**Affiliations:** 1 Pharmaceutical Commercialization Technology, MMD, Merck & Co., Inc., Rahway, NJ 07065, USA; robert_meyer2@merck.com; 2 Process Analytical Technology, Animal Health, Merck & Co., Inc., Rahway, NJ 07065, USA; jennifer_potter@merck.com

**Keywords:** film coating, optical coherence tomography, X-ray computed tomography, film thickness

## Abstract

**Background/Objective:** Film coatings are vital components of many pharmaceutical products consumed orally in solid dosage form, and the optimization of the film coating unit operation is critical to the success of these products. It is essential to maintain adequate film coat thickness on tablets to ensure the elegance, mechanical integrity, and controlled-release functionality of active pharmaceutical ingredients. We aim to evaluate techniques for measuring the film coat thickness of tablets in the pharmaceutical drug product development space as current research primarily focuses on in-line methods at the manufacturing scale. **Methods:** A total of four tablet types, varying in size, shape, and coating thickness were assessed using Optical Coherence Tomography and X-ray Computed Tomography. The data was then compared to baseline reference values gathered by examining tablets with a Confocal Microscope. A second trial was performed using an alternative Optical Coherence Tomography instrument to verify the accuracy of the data. The methods were also evaluated on additional criteria utilizing a Pugh Matrix. **Results**: The initial Optical Coherence Tomography yielded measurements that were inconsistent with the values provided by the control for three of the four tablet types; however, the follow-up study provided values within an acceptable range. The X-ray Computed Tomography also provided accurate measurements but presented challenges for precision in relation to the instrument’s resolution capabilities. Based on the assessment of selected criteria, Optical Coherence Tomography is ideal for all clear-coated tablets, while X-ray Computed Tomography is better suited for small tablets with either opaque or clear coats. **Conclusions:** Optical Coherence Tomography, X-ray Computed Tomography, and the use of a Confocal Microscope are all viable methods for measuring the film coat thickness of tablets. Method selection is not absolute and depends on factors such as safety, ease of use, adaptability, and tablet characteristics. The results of this study will help provide guidance for selecting the most appropriate method for measuring the film coat thickness of a specific tablet.

## 1. Introduction

Film coating is the process of spraying a thin, uniform, polymer-based coating on the surface of an oral solid dosage form [1]. Tablets are placed in a rotating pan where spray guns will release atomized droplets containing a blend of polymers, pigments, and plasticizers suspended or dissolved in a solvent that will spread over the surface and leave a deposited material coating the tablets. The tablets continuously tumble through the spray zone and hot air to build up and dry the coating [2]. There are various film coating techniques and parameters that can be optimized based on the type of tablet, suspension properties, and coating scale [3].

There are two distinct classes of film coating: nonfunctional and functional. Nonfunctional coatings are used to alter appearance and improve the elegance of the tablet. The coating can also mask the taste, enhance mechanical strength, improve active pharmaceutical ingredient (API) stability, and protect tablets from humidity, oxidation, and light effects. Functional coatings can offer the same benefits as nonfunctional coatings in addition to modifying or delaying drug release [1].

Understanding tablet film coat thickness is critical for ensuring proper scale-up and coating functionality. The coating unit operation is transferred from the lab to the manufacturing scale using a variety of scale-independent and dependent factors as well as modeling, and it is important to verify that coating structure and thickness uniformity are maintained in the process [4]. Inconsistencies can negatively impact the protection of the tablet, the tablet’s elegance, dissolution behavior, and, in the case of functional coatings, the controlled release of the API. While the drug product is in the development stage, the film coating process is optimized, and it is essential to verify that the target film thickness is achieved and can be consistently reproduced in subsequent batches. However, with evolving technology, there are no established methods for accurately and precisely measuring film coat thickness while also assessing the quality of the coating.

In the case of functional coatings, release behavior directly corresponds to coating thickness, and there is motivation from the U.S. Food and Drug Administration to implement new monitoring and process-control methods that replace the existing empirical approaches to gain further understanding of the pharmaceutical manufacturing process [5]. This initiative is seen within the Process Analytical Technology field through the evaluation of existing tools and instrumentation capable of measuring film coat thickness, specifically at the lab scale.

There are various existing methods for measuring tablet film coat thickness at commercial scale that utilize in-line batch monitoring. Two of the most common techniques are Near-Infrared Spectroscopy (NIR) and Raman Spectroscopy [6]. These methods require process-specific calibration models that are set based on sample and batch size [7]. These methods present challenges at the lab scale where the film coating process has not yet been developed or is still under development and has varying batch sizes. Despite the advancements of in-line monitoring technologies, there remains limited understanding of how to accurately measure film coat thickness on individual tablets or small batches at the pilot and lab scales. This is further complicated by the presence of multiple drug products with distinct characteristics within a facility, which necessitates frequent instrument recalibration for each tablet type, which overall reduces measurement efficiency and consistency.

Another common analysis technique is Scanning Electron Microscopy (SEM). Although SEM can be used to scan and measure the film coat thickness of individual tablets, it also requires time-consuming sample preparation and is a destructive technique that can present exposure hazards when working with Occupational Exposure Band (OEB) 4/5 products. There are new advances detailing non-destructive SEM techniques; however, this method is reserved for in-line batch monitoring [8]. Due to equipment availability restraints, SEM evaluation was excluded from the scope of this study.

As this study works with individual tablets rather than batches, the Keyence, a 3D Laser-Scanning Confocal Microscope, was selected as the baseline reference method for measuring tablet film coat thickness. The current industry standard for regulatory filing of film coat thickness is tablet weight gain, which is performed by comparing the pre-coated weight of the core tablet to the tablet weight after coating using a percent change. Where established, the quantity of coating weight can be associated with a film coat thickness [6]. This approach is limited in its applicability as a reference method for precision and accuracy as it does not yield exact thickness values. Although predictive models exist that estimate film thickness from percent weight gain by utilizing coating solution density and tablet surface area, these models only provide estimated thickness values and fail to account for the inherent variability from the coating process. The percent weight gain method also does not provide any information regarding the quality or uniformity of the film coat. Given the limited research on analytical techniques for individual tablet coating thickness measurements at the lab scale, no universally accepted analytical method currently exists for this specific application. As a result, the Keyence Microscope was selected as the reference standard for evaluating precision and accuracy. This instrument offers high-resolution visualization of the film coat, enabling direct imaging of the cross-sectional region of a tablet. Thickness measurements were performed manually using the microscope’s integrated caliper software tool on captured images. The measurements recorded using the Keyence were able to be directly compared to the measurements recorded by the other instruments as the same samples were used for all three methods.

Optical Coherence Tomography (OCT) is a noninvasive imaging tool traditionally used within Ophthalmology to view different layers of the retina [9]. OCT utilizes beams of near-infrared light to scan a sample and then measures the depth of the light that is back-reflected to create a cross-sectional image of the sample layers and structures [10]. The OCT used in this study was a spectral-domain instrument that contained a light source with broad optical bandwidth and a high-sensitivity spectrometer for better signal-to-noise ratio detection [11]. One key element of OCT is that the light beams must pass through a material’s surface to effectively reflect and obtain a measurement. Therefore, the OCT can only be used on materials that allow light transmittance. Additionally, since light travels differently through various materials, OCT relies on refractive index values to accurately measure sample thickness and properly interpret the resulting image [12]. Although OCT is an established system, there are limited research applications of OCT in the pharmaceutical industry. Previous research has demonstrated feasibility of using OCT for in-line batch monitoring and methods of optimization [13], but this study aims to examine the efficacy and constrains of this technique for quantifying film coat thickness during the drug product development phase.

X-ray Computed Tomography (XRCT) is a noninvasive technology typically used in the medical field for diagnostic imaging. A narrow beam of X-rays is aimed and rotated around a sample that analyzes the attenuation of the beam through the different densities to generate cross-sectional images, or slices. These slices can then be reconstructed and stacked together to form a three-dimensional image of the sample that shows the internal structures [14]. A microscopic version of XRCT is commonly used in the pharmaceutical industry to analyze the morphology and internal characteristics of tablets [15]. There are various feasibility studies supporting the use of XRCT to analyze the coating thickness, uniformity, porosity, and density of tablets [16]. While this technique is more developed for pharmaceutical use than OCT, the study aims to assess its limitations and adaptability for tablets of varying size and coating thickness.

## 2. Materials and Methods

Four different tablets were utilized in this study and were examined using three different analytical methods: Keyence Microscope, OCT, and XRCT. The tablets were selected to encompass a range of film coat thicknesses and coating formulations. Although selection was not based on specific tablet geometries, the sample set represents common tablet shapes encountered in the pharmaceutical industry. Tablet dimensions, shapes, and coating formulations are summarized in Table 1. Due to the inherit limitations of the OCT, all tablets included in this study possessed a clear film coat to ensure measurement feasibility. All tablets were coated in a pan coater, and, with the exception of Tablet B, all tablets were placebos. Sample sizes for each method can be found in Table 2. The tablets used in this study were supplied by Merck & Co., Inc., Rahway, NJ, USA.

The first analytical method assessed was Optical Coherence Tomography using the Wasatch Spark OCT from Wasatch Photonics, Morrisville, NC, USA. The equipment had pre-set parameters that remained consistent between tablets and can be found in Table A1. As this OCT had a camera for visualization, the tablet bands were marked to ensure four different regions were measured on each tablet. Tablet A was analyzed first, and each tablet was measured individually, with two to four measurements recorded per region based on image clarity. Measurements were taken using the OCT Software’s v2.1.8.0 integrated caliper tool. This tool was used to draw a line from the outside edge of the tablet’s film coat to the interface of the tablet’s core, and the software provided a measurement in the magnitude of microns based on the length of the line. This process was repeated for Tablets B, C, and D. The only adjustments made between tablet types were the manual repositioning of the tablets under the source and the focus, which was adjusted by shifting the height based on the thickness of the tablet. Each tablet was mounted to the base of the OCT using double-sided carbon tape to prevent the tablet from shifting. A custom covering was also designed to shield the laser and tablet from ambient light to limit light interference within the images. The tablets were individually bagged and labeled to ensure consistency with other tablet measurements.

To accurately interpret the measurement results, a refractive index must be applied. The Wasatch Spark OCT does not have internal capabilities to measure the material’s refractive index, so the coating solutions for Tablets B and D were measured using a refractometer (ATAGO PAL-RI, ATAGO USA, Inc., Bellevue, WA, USA). Three drops of the liquid coating solutions were placed on the refractometer and allowed to dry. Measurements were recorded at regular intervals until a dry film was achieved, providing a representative reading of the refractive index of the solutions in the solid state. These trials were completed three times for both solutions. The end values were averaged, and the corresponding refractive indices were used to perform the final calculations for the OCT measurements for each tablet. Tablets A and C were coated with the same coating solution and the refractive index values were extracted from previous film-coat related program testing. The refractive index of each coating solution can be found in Table 3.

In parallel with the OCT measurements, XRCT scans and measurements were performed utilizing the Bruker SkyScan 1275 (Bruker, Billerica, MA, USA). Parameters were optimized for each tablet type and settings can be found in Table A2, Table A3, Table A4 and Table A5. Each tablet was mounted to the brass stand using double-sided carbon tape and individually scanned using the SkyScan Scanning Control software v1.7. Images were reconstructed in the NRecon reconstruction software v1.7.4.6 using GPUReconServer V1.7.4.2 and analyzed using DataViewer v.1.5.6.2. Following a similar process to the OCT, measurements were taken in each of the four quadrants at three layers of the tablets for a total of 12 data points per tablet. The coating thickness was measured using the DataViewer’s integrated caliper tool. A line was drawn from the outside edge of the film coat to the interface of the film coat and the tablet core. The software then provided a measurement of pixels that was automatically converted to microns using the corresponding image resolution. The software’s attenuation graph was also utilized to help verify the caliper length in regions where the delineation between core and film coat was not clear. The sample sizes for each tablet can be found in Table 2. Due to the size of Tablet A, it was cut through the center debossing and attached by the face to the stand in order to maximize the resolution of the image.

The final method utilized a Keyence VK-X1000 Laser Microscope (Keyence Corporation, Elmwood Park, NJ, USA). This is a destructive technique and required cutting of each tablet using a tablet cutter under a ventilated balance enclosure and was therefore performed last to ensure that the same samples could be used for each of the three analytical methods. The tablets were bisected along the center band and positioned on the base of the Keyence to enable cross-sectional imaging through the microscope camera. Using best practice microscopy techniques, the tablets were brought into the field of view and focused between 10× to 20× magnification. Following a similar process to the OCT and XRCT, each tablet was measured individually with three to five measurements recorded for each region of the band of the tablet over four quadrants. The measurements were performed using the Keyence software’s (v.1.1.3.184) built-in caliper tool and a line was drawn from the outer edge of the film coat to the interface of the film coat and tablet core. This provided a direct measurement of the film coat on the order of microns.

A second study was performed by a separate Merck & Co., Inc., Rahway, NJ, USA team using the OSeeT Pharma 1D by Phyllon GmbH. This OCT has the capacity to provide automatic film coat measurements of multiple tablets at once. This subsequent study was performed in an OEB 4/5 designated isolator fume hood using non-GMP Tablet D samples. A total of 129 tablets were analyzed, and the data was provided to compare with this study.

All data was recorded in Electronic Laboratory Notebook Microsoft Excel Spreadsheets and data analysis was performed using Minitab Statistical Software (Minitab 22). In order to reduce variability between methods, one individual performed the measurements across all tablets and instruments and was adequately trained on the execution of each method. To evaluate additional factors impacting method performance, an Analytical Hierarchy Process was used to assign weights to selected criteria. These weights were then applied in a Pugh Matrix to score each method based on its performance in the respective categories.

## 3. Results

### 3.1. Tablet Trial Measurements

#### 3.1.1. Keyence Microscope

**Figure 1:**
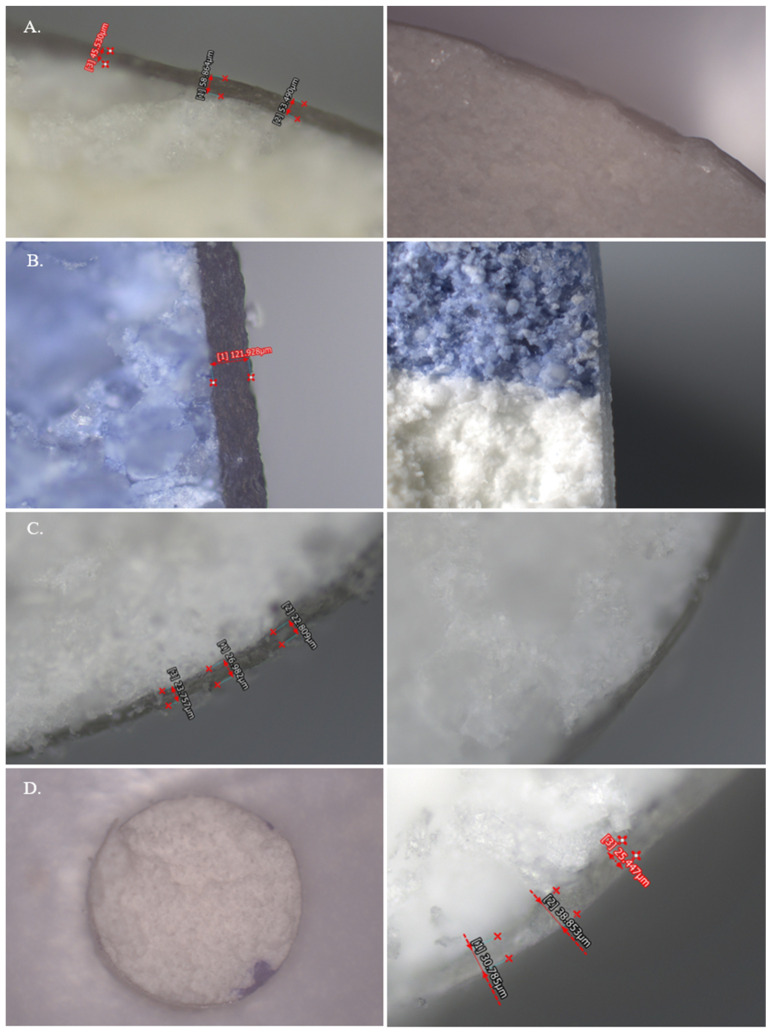
Images captured via the Keyence Microscope: (**A**) Tablet A; (**B**) Tablet B; (**C**) Tablet C; (**D**) Tablet D.

The Keyence Microscope was used to perform baseline reference measurements of the physical tablet film coat. Figure 1 shows the images produced by the Keyence for each tablet. Tablet B presented with the thickest film coat with an average thickness of 142.0 µm (*n* = 3). Tablet A contained the next thickest film coat with an average of 40.9 µm (*n* = 3), and Tablet D followed with an average thickness of 29.0 µm (*n* = 12). Tablet C contained the thinnest film coat with an average thickness of 24.9 µm (*n* = 6) as presented in Table 4. When looking at the variability of the measurements, Tablet C had the largest RSD (25.7%) while Tablet B had the lowest RSD (9.2%). The average RSD among all four tablets was 18.1%.

#### 3.1.2. Optical Coherence Tomography

**Figure 2:**
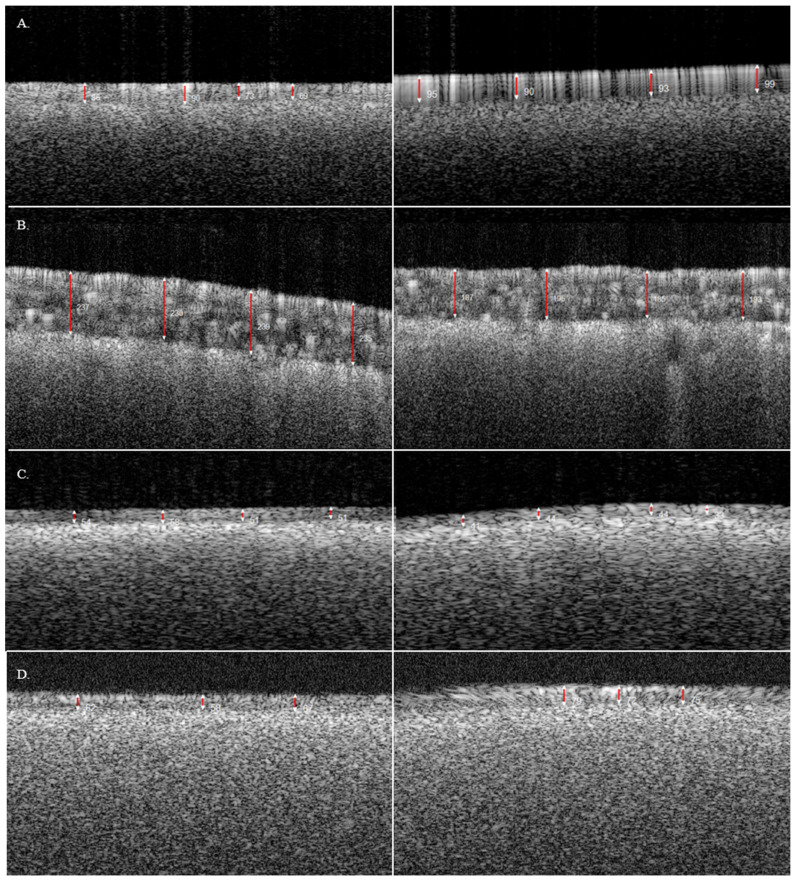
Images captured via Optical Coherence Tomography: (**A**) Tablet A; (**B**) Tablet B; (**C**) Tablet C; (**D**) Tablet D. Red line indicated caliper measurement.

OCT was then used to measure the tablet film coat thickness for the bands of all four tablets. Figure 2 shows the images generated using OCT for each tablet. Similarly to the Keyence, Tablet B had the thickest coating which measured 135.9 µm (*n* = 6). Tablet A and Tablet D then followed with an average thickness of 61.3 µm (*n* = 6) and 46 µm (*n* = 12), respectively. Tablet C also had the thinnest coating which measured 33.6 µm (*n* = 6) as shown in Table 5. Compared to the Keyence measurements, the OCT values followed the same trend in overall coating thickness; however, all mean values differed from the Keyence values. Tablet A shows a 49.9% increase in thickness, while Tablet B showed a 2.4% decrease in thickness. Tablets C and D also showed a measurement increase from the of 35.0% and 58.6%, respectively, compared to the Keyence values. The OCT generated values with lower variability compared to the Keyence, with an average RSD of 11.5% across all four tablets. Tablet B also had the lowest RSD of 6.0% while Tablet A had the highest variability with an RSD of 15.3%.

#### 3.1.3. X-Ray Computed Tomography

**Figure 3:**
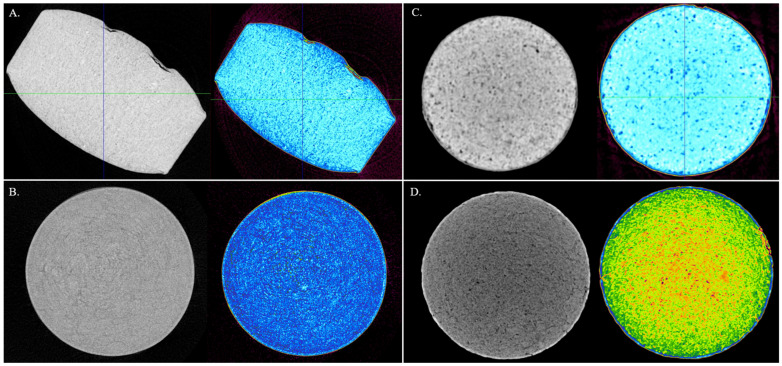
Images captured via X-ray Computed Tomography: (**A**) Tablet A; (**B**) Tablet B; (**C**) Tablet C; (**D**) Tablet D. Images are shown in grayscale and color-scale to visualize film coat and core interface.

XRCT was performed to measure the film coat thickness of the band of all four tablet types. Figure 3 shows the images gathered by the XRCT for each tablet. Following a similar trend as the Keyence, Tablet B had the thickest average film coat measuring 142.1 µm (*n* = 1). Tablet C also had the smallest average thickness measuring 23.3 µm (*n* = 6). Tablets A and D measured 52.0 µm (*n* = 3) and 30.1 µm (*n* = 12), respectively (Table 6). Like the OCT, Tablet A showed the highest RSD of 27.1% and Tablet B showed the lowest RSD of 12.3%. The XRCT showed the highest variability across all three methods with an average RSD of 20.0%. Tablets B, C, and D all yielded closer mean values than the OCT to the Keyence, however Tablet A had the greatest difference from the Keyence with a 27.1% increase in mean thickness measurement.

### 3.2. Data Analysis

**Figure 4:**
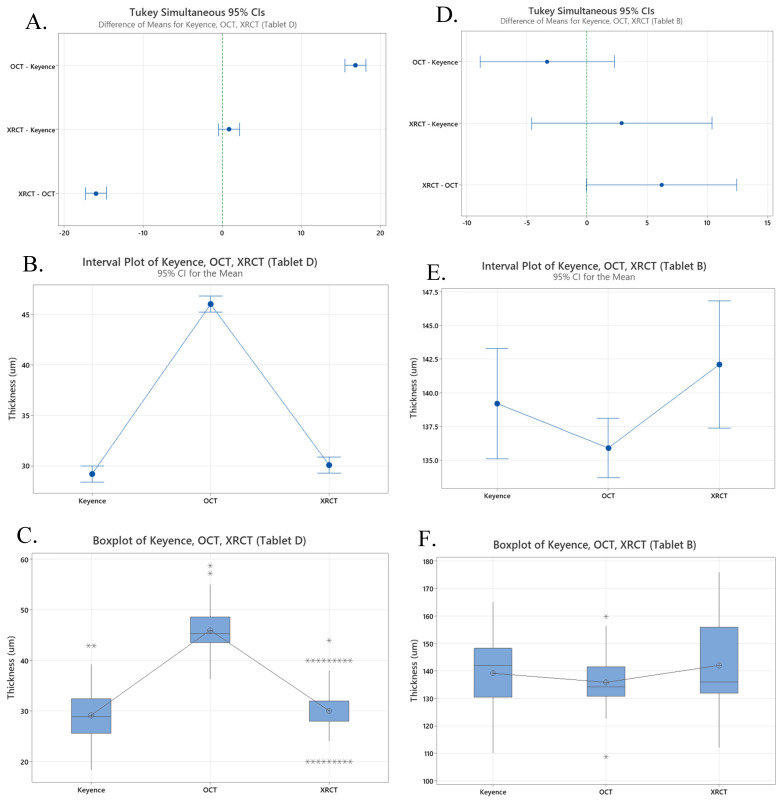
Data plots: (**A**) Tukey simulation with 95% confidence interval comparing Keyence, OCT, and XRCT data for Tablet D; (**B**) interval plot comparing Keyence, OCT, and XRCT data for Tablet D; (**C**) boxplot comparing Keyence, OCT, and XRCT data for Tablet D; (**D**) Tukey simulation with 95% confidence interval comparing Keyence, OCT, and XRCT data for Tablet B; (**E**) interval plot comparing Keyence, OCT, and XRCT data for Tablet B; (**F**) boxplot comparing Keyence, OCT, and XRCT data for Tablet B.

A one-way ANOVA was initially performed in Minitab using data from Tablet D, which had the largest number of data points collected across the three (*n* = 144). The analysis revealed a *p*-value of 0.00 when comparing the Keyence, OCT, and XRCT. Paired two-tailed *T*-tests were also performed comparing the values for Tablet D for the Keyence and OCT and the Keyence and XRCT (Table 6). Figure 4A shows the Tukey analysis which indicates that the Keyence and XRCT can be in the same group, group B, with 95% confidence intervals (28.4, 30.0) and (29.3, 30.9), respectively. The interval plot and boxplot in Figure 4B,C also show the overlapping groups of Keyence and XRCT measurements, while the OCT confidence interval is (45.2, 46.8).

Further data analysis was conducted using Tablet B, due to its significant size difference compared to Tablet D. Performing a one-way ANOVA in Minitab using Tablet B comparing Keyence, OCT, and XRCT revealed a *p*-value of 0.043, which is less than the designated α = 0.05. Paired two-tailed *T*-tests were performed comparing the values for Tablet B for the Keyence and OCT and the Keyence and XRCT (Table 7). The Tukey analysis shown in Figure 4D reveals that all three methods can be grouped with the following 95% confidence intervals: (135.1, 143.28), (133.7, 138.1), and (137.4, 146.8). The interval plot and boxplot in Figure 4E,F also show the overlapping groups of the Keyence, OCT, and XRCT measurements.

### 3.3. External OCT Study

**Figure 5:**
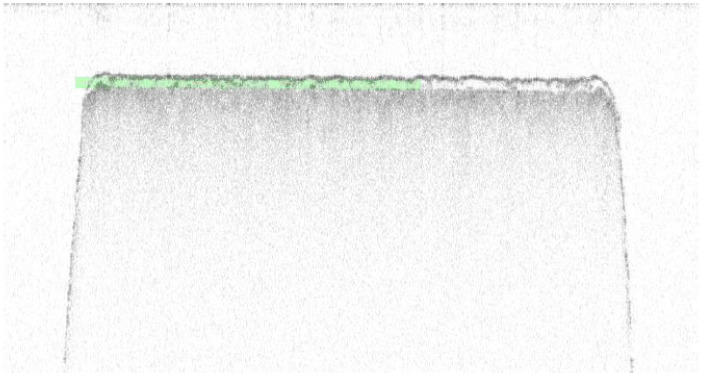
Image captured via OSeeT Pharma 1D of Tablet D. Green shading highlights the film coat region of the tablet.

**Figure 6:**
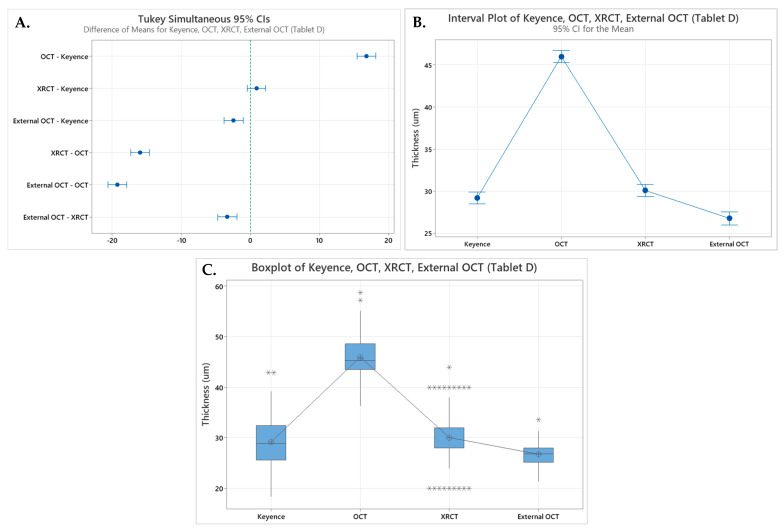
Data plots: (**A**) Tukey simulation with 95% confidence interval comparing Keyence, OCT, XRCT, and external OCT data for Tablet D; (**B**) interval plot comparing Keyence, OCT, XRCT, and external OCT data for Tablet D; (**C**) boxplot comparing Keyence, OCT, XRCT, and external OCT data for Tablet D.

A follow-up study was conducted for Tablet D (n = 128) using an alternative OCT instrument. Figure 5 shows an image captured by the OSeeT Pharma 1D by Phyllon GmbH of Tablet D. Using a total of 128 tablets yielded an average film coat thickness of 26.4 ± 2.8 µm with an RSD of 10.61%. The OSeeT value has a 54.1% decrease from the original OCT method, and a decrease of 8.9% and 12.3% from Keyence and XRCT, respectively. A paired two-tailed *T*-test comparing the OSeeT data to the Keyence yields a *p*-value of 0.035. The Tukey analysis (Figure 6A) also reveals that the OSeeT falls in its own grouping despite being closer in value to the Keyence and XRCT than the original OCT method using the Wasatch Spark OCT. The interval and box plot are also shown in Figure 6B,C. With a 95% confidence interval from (25.9, 27.5) the interval plot does not show overlapping; however, the box plot reveals greater overlap with the Keyence and XRCT than the original OCT.

## 4. Discussion

### 4.1. Measurement Precision and Accuracy

The Keyence Microscope was utilized as a baseline tool to compare measurements from OCT and the XRCT, as it produces direct images of tablets and film coat while OCT and XRCT generate reconstructed images. Although the Keyence method aims for precision and accuracy, a reasonable standard deviation is expected due to the inherent variability of the film coat. This variability is a normal factor resulting from the overall film coating process and can cause differences in thickness both across the surface of individual tablets and among tablets from the same batch [17]. The uneven coating visualized through the Keyence supports the high relative standard deviation values. Additionally, across all methods, Tablets A and C consistently exhibited the highest RSD values while Tablet B showed the lowest. However, the mean and median values presented in Table 4 are closely aligned, indicating a symmetric distribution and supporting the use of the Keyence Microscope as a reference method for film coat thickness measurements.

When comparing the OCT and Keyence values in Figure 4B,C for Tablet D, there is no overlap in the interval pots and two outliers overlapping the boxplot within the 50% middle region. These plots combined with the results of the *T*-test yielding a *p*-value of 0.0 indicate that there is strong evidence differentiating the measurements between the Keyence and the OCT. However, when looking at the comparisons for Tablet B in Figure 4E,F there is an overlap between the data for the Keyence and Wasatch Spark OCT. Additionally, the *T*-test comparing the data for Tablet B measured by the OCT and Keyence yields a *p*-value of 0.207, which is greater than α= 0.05, indicating that there is no statistical difference between the data sets. The results of the data analysis show that the OCT did not provide accurate film coat thickness measurements; however, the measurements were the most precise out of all three methods with the lowest average RSD of 11.5% for all four tablets.

This poses the question of the accuracy of the OCT as there is a statistically significant difference between the data for Tablet D measured by the OCT and Keyence, and no statically significant difference for the measurements of Tablet B. After investigating the data and looking at the results of Tablet A and C, it was hypothesized that an X-factor could be used to adjust the data as the values were precise, but not accurate. To attempt to calculate an X-factor, the following procedure was followed:

(1) $$X\text{-}\mathrm{Factor}=\frac{Post\;Refractive\;Index\;Calculated\;Value}{Mean\;Keyence\;Value}$$

**Figure 7:**
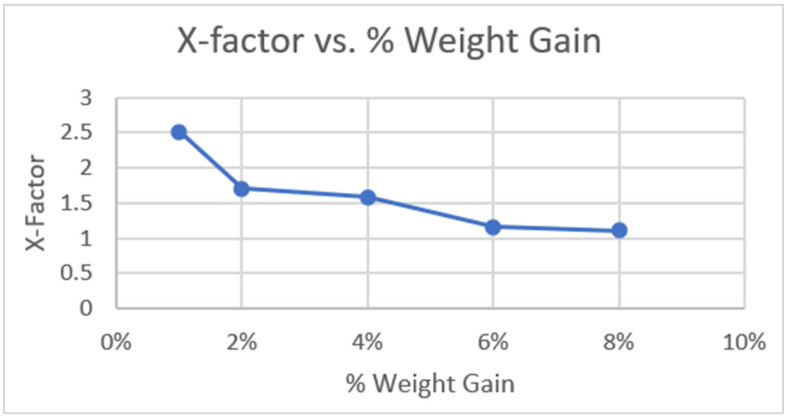
Graph of X-factor vs. percent weight gain showing the decrease in X-Factor as film thickness increases for the Wasatch Spark OCT.

The generated X-factors for Tablets A–D were determined to be 1.39, 0.96, 1.41, and 1.58, respectively. These values are inconsistent and when looking at the nominal thickness of the tablets generated by the Keyence, there is a correlation between thickness and X-factor adjustment. The thicker the film coat, the less adjustment is needed, and the X-factor decreases as thickness increases as shown in Figure 7, where thickness is presented as percent weight gain. These values were obtained from an additional experiment measuring Tablet A coated at increasing thickness and examined using the Wasatch Spark OCT. This is further supported by the accurate OCT measurements of Tablet B, which had the thickest film coat and showed the smallest difference from the reference value, with only a 2.4% difference. As a result, a set X-factor value could not be determined, and further testing is needed to generate a model that is representative of the necessary adjustment factor.

The exact reason for the inaccuracy of the thickness values generated from the Wasatch Spark OCT is unknown and needs to be further investigated. However, it is believed that the adjustment is isolated to the specific OCT and is not applicable to all OCT instruments.

**Figure 8:**
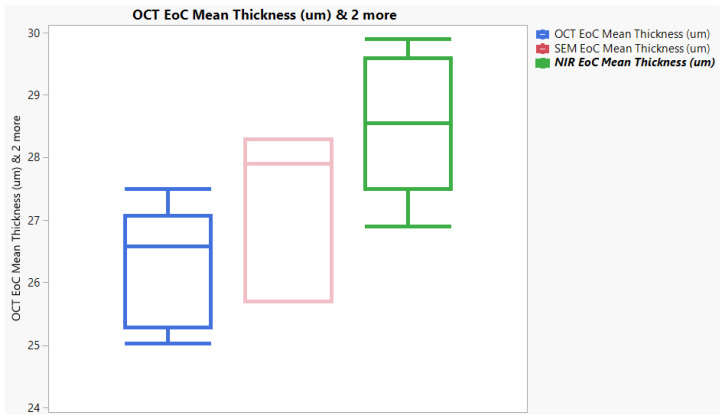
Comparison of OSeeT mean thickness data at end of coating to qualified NIR and SEM thickness measurement data for Tablet D.

Due to the inconsistencies present in the OCT data using the Wasatch Spark OCT, a second OCT study was conducted with an alternate OCT instrument, the OSeeT by Phyllon GmbH, using Tablet D. This data yielded a mean of 26.4 µm, which is a 54.1% difference from the mean of 46 µm that was gathered from the Wasatch OCT. The plots presented in Figure 6B,C show a greater overlap of the Keyence and OSeeT OCT data than the Wasatch Spark OCT data, although the data still falls in different groupings. The *T*-test comparing the Keyence to the OSeeT OCT data also yielded a *p*-value of 0.035 which is less than α = 0.05, indicating a statistically significant difference. However, this is expected as the second OCT study utilized Tablet D samples from a different batch than the Keyence. The OSeeT OCT study provides closer data to the Keyence and provides more support for the OCT as an acceptable method of accurately measuring tablet film thickness. Additionally, the data obtained from the OSeeT was compared to SEM and NIR which are qualified methods utilized by the team that performed the second OCT study (Figure 8). With the significant overlap of the data from the validated SEM method, there is further support for OCT as an accurate and reliable method. However, due to the variability between the data sets, further experiments should be conducted to definitively establish the accuracy of the OCT method for measuring tablet film coat thickness.

XRCT was also used to measure the film coat thickness of the four different tablets. When comparing the XRCT to the Keyence values as seen in Figure 4B,C, for Tablet D there is overlap present in both the interval plot and boxplots. However, the boxplot does reveals both high and low outlier values, and the XRCT had the highest RSD average across the four tablets indicating issues with the precision of the measurements. These plots, combined with a *p*-value of 0.135 generated from a two-sample *T*-test indicate that there is no statistically significant difference between the measurements generated from the Keyence and XRCT for Tablet D. Similarly, when looking at the analysis of Tablet B in Figure 4E,F, there is again overlap of the interval and boxplots, with fewer outliers than Tablet D. A *p*-value of 0.525 was generated from a two-sample paired *T*-test, which is greater than the indicated α = 0.05, also supporting that there is no statistically significant difference between the Keyence and XRCT for Tablet B. The data analysis reveals a strong indication of accurate measurements from the XRCT but combined with the large relative standard deviations, raises questions regarding the precision of the measurements.

An ANOVA test was performed comparing all three methods for Tablet B and D which yielded a *p*-value of 0.043 and 0.0, respectively, both of which are less than the designated alpha value of 0.05, indicating that there is evidence to reject the null hypothesis that all three instruments provide the same measurement. However, this rejection is expected considering the X-factor adjustments needed for the OCT. When looking at the OCT, used in the primary study, and XRCT individually compared to the Keyence measurement data, the OCT provides precise, but inaccurate data, whereas the XRCT provides accurate but imprecise data.

### 4.2. Pugh Matrix

**Figure A1:**
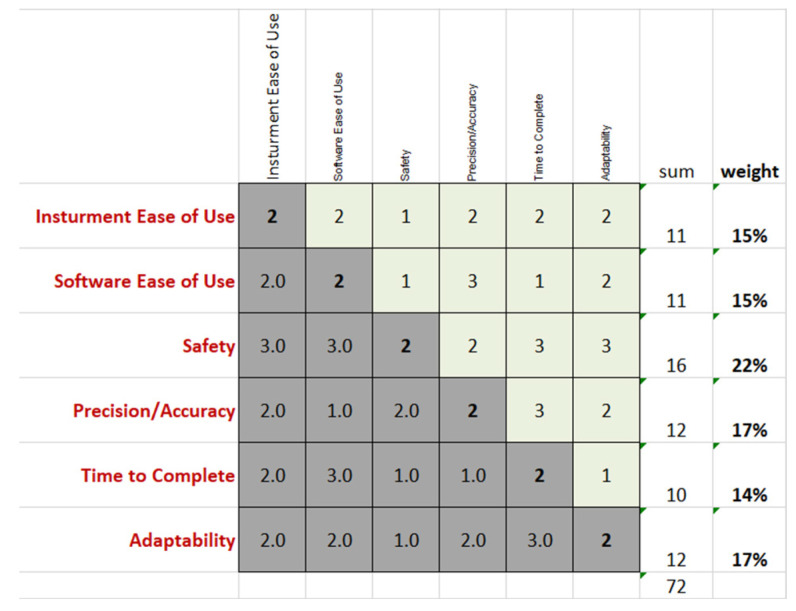
Analytical Hierarchy Process showing weighting for selected criteria of instrument ease of use, software ease of use, safety, precision/accuracy, time to complete, and adaptability.

**Figure A2:**
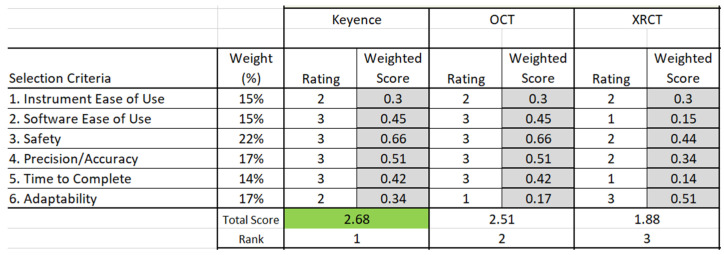
Pugh Matrix using weighted selection criteria to score the Keyence, OCT, and XRCT on a scale of 1–3.

While precision and accuracy are fundamental criteria for evaluating the efficacy of measurement methods, additional factors must be considered when selecting an instrument for film coat thickness measurement during the development stage. These factors include, but are not limited to, ease of use, safety, and adaptability. An Analytical Hierarchy Process was employed to determine the relative importance of the selected criteria. Each criterion was systematically compared to the others to establish weighting factors that were subsequently incorporated into a Pugh Matrix, which was used to evaluate and compare the performance of the three measurement methods. The Pugh Matrix utilized a scoring scale ranging from 1 to 3, where 1 indicates the lowest performance and 3 the highest, for each criterion. This structured approach enabled a comprehensive comparison of the OCT and XRCT methods relative to the Keyence Microscope. The complete Analytical Hierarchy Process and Pugh Matrix can be found in Figure A1 and Figure A2, respectively.

The selected criteria include instrument ease of use, software ease of use, safety, precision/accuracy, time to complete, and adaptability. Stakeholders in the oral solid dosage development space were consulted to help develop these criteria and associated weighting. GMP compliance is not listed as a criterion for the lab/development scale as development labs are commonly non-GMP facilities; however, methods used at manufacturing sites must adhere to GMP compliance. Additionally, cost was not factored into the criteria ranking as at the time of testing, all methods were available in the utilized lab-space. Acquiring a new instrument will necessitate vendor research and depend on the desired design and complexity, as there are a multitude of options available on the market.

Within the Analytical Hierarchy Process, safety was determined to be the most critical criterion. When comparing the methods, the only safety concern for the OCT is the use of the laser, but the instrument has an inherently safe design and should not pose any significant risks. Both the Keyence and the XRCT presented minor safety considerations. The Keyence method requires physical destruction of tablets and is therefore only recommended for OEB 1–3 products that can be safely handled and cut within a ventilated balance enclosure. In contrast, the XRCT method is non-destructive but involves possible radiation exposure, necessitated specialized training and dosimeter usage for operator safety. Therefore, the Keyence and the XRCT were given the same score, and the OCT received the highest score.

Precision and accuracy were valued criteria as the method must provide measurements that are representative of the true film coat thickness of the tablet. As previously described, the Keyence served as the trusted reference measurements, with the only variability resulting from the inherent nonuniform coating thickness on the surface of tablets, and non-automated measurements, but both factors are consistent across all three methods. The OCT yields precise data with the lowest relative standard deviations, although the accuracy of the specific instrument was significant due to possible miscalibration. This was resolved with the presentation of accurate data via the External OCT Study and therefore received the same score as the Keyence as the inaccuracies are expected to be isolated to the specific OCT instrument used in the primary tablet trials. The XRCT presented the lowest precision and accuracy due to the resolution limitations, especially with larger tablets. Tablet B was the largest tablet and therefore could only reach a resolution of 7 µm/voxel based on distance from the source, but the instrument has a maximum resolution of 4 µm/voxel. The XRCT could be sufficient for thicker film coats (>50 µm) but can present challenges for thinner film coats or large tablets (>4 mm), especially in the case of functional coatings where a set thickness needs to be validated.

Adaptability received the same priority ranking as precision and accuracy. When working in the drug product development stage, the spray process is being optimized, so it is necessary to check coating thickness to observe how the film is building for a set of process parameters. Additionally, multiple distinct types of tablets and film coats are commonly in the development pipeline and present in a laboratory space at a given time, so the method needs to be versatile and have the capacity to measure a variety of tablets. This is in comparison to the manufacturing scale where in-line film thickness measurement tools are typically calibrated to a specific product with confirmed process parameters.

All three methods have unique challenges with adaptability. The Keyence does not have any size limitations and can visualize all varieties of film coats, regardless of thickness, including both clear and opaque coats. However, Keyence measurement requires the physical cutting of a tablet that exposes the core and is therefore limited to non-potent products. It is a possibility that the Keyence can be integrated into an isolator to measure OEB 4/5 tablets, but this would increase challenges with ease of use and safety. The OCT also does not have any size or thickness limitations, although thicker coatings can be observed more distinctly. However, the OCT can also only be used for clear-coated tablets as the light cannot penetrate through opaque coatings. The OCT is also non-destructive and is therefore a safer option if needed for potent products. The XRCT has minor size and thickness limitations due to its resolution capabilities, but it is important to note that all four tablets used in the study, which varied in size and thickness, were successfully measured using XRCT, although the image quality was affected. Additionally, the XRCT can measure both clear and opaque film coats, and like the OCT, is a non-destructive method.

When ranking adaptability, product portfolio consideration is crucial; if there are no potent products, then the Keyence is the most adaptable, and if there are only opaque products, then the OCT cannot be used. An additional consideration for adaptability is the multipurpose use of the instrument. Versatility enhances the overall value of the instrument by allowing it to address multiple analytical needs within the development process, thereby improving efficiency and reducing cost by limiting the need for multiple specialized devices. For instance, the XRCT is traditionally used to evaluate internal defects, such as cracking and the Keyence also has a laser functionality which measures surface roughness. However, as OCT was not originally designed for pharmaceutical use it is therefore limited in its applications in a development lab. Without specific product portfolio considerations, the XRCT received the highest score as size and thickness primarily impact image quality and there are no hard limitations, but these scores can vary for individual use cases.

Instrument and software ease of use are also criteria to evaluate when considering implementing a new method. For this study, both software and instrument were evaluated separately, but hold the same scoring weight. The OCT and Keyence systems exhibit comparable ease of use featuring straightforward interfaces and simple adjustments for visualizing the film coat and capturing images. Both methods require positioning the tablet under the camera, focusing the image, and using the built-in caliper tool to manually measure coating thickness. The Keyence provides direct imaging but necessitates physical cutting of the tablets. Once a tablet is secured on the stand, it remains stationary and can be shifted using the microscope controls to bring a region into the field of view. However, at high magnification, focusing the camera can be challenging, requiring frequent adjustments and repositioning to obtain multiple measurements. Alternatively, the OCT requires manual rotation of the tablet to capture images of different quadrants and similar focus adjustments to achieve a clear reconstruction image. The OCT software v.2.1.8.0 allows the image to be frozen, enabling multiple measurements to be recorded simultaneously. A second camera view also facilitates precise adjustment of the laser and identification of the measurement point to help improve visualization of the tablet film coat. Additionally, the measurements generated on the OCT are not the true measurements and require a calculation using the refractive index of the coating solution, which may need to be found separately if not already known. Some OCTs, such as the OSeeT used in the follow-up OCT study, have a feature that automatically performs calculations to provide final measurement data, whereas, for the Wasatch OCT, the data had to be extracted and calculated separately.

In terms of ease of use, the XRCT requires minimal set-up beyond mounting the tablet within the scanner. However, the overall process is more complex compared to other methods. This study utilized three software programs associated with the XRCT system: SkyScan 1275 Scanning Software v.1.7, NRecon v.1.7.4.6, and DataViewer v.1.5.6.2. The most challenging aspect of the XRCT is optimizing the scanning parameters which vary depending on the tablet’s size and density. Parameters such as voltage, exposure time, scanning angle, and resolution must be carefully adjusted for tablet type to achieve optimal image quality. Following the scanning procedure, image reconstruction is required, involving additional parameter adjustment to enhance image clarity. The DataViewer software v.1.5.6.2 provides multiple viewing options to visualize the tablet from different orientations. The primary limitation of DataViewer is that the built-in caliper tool allows only measurement to be recorded at a time. However, it offers an attenuation graph that aids in distinguishing the interface between the core and the film coat with the delineation is unclear. Considering these factors, both the Keyence and OCT received equivalent scores for instrument and software ease of use, while the XRCT while assigned a lower score.

The final criterion to consider is the time to complete. At the development stage, the goal is to obtain a quick measurement to verify parameters and coating thickness as the measurements may need to be taken multiple times for multiple tablets. The Keyence and OCT only allow one tablet at a time to be measured and took approximately the same duration to complete. However, the OCT used in the second study had the capacity to measure multiple tablets at once, which based on the specific instrument can decrease the testing time. With normal XRCT use for internal defects, multiple tablets can be contained and measured in one scan, but due to the need to bring the tablet as close to the source as possible, only one tablet could be scanned at a time. With the set scanning parameters, each scan took approximately two hours to complete and an additional 15 min for reconstruction. Although time-consuming in comparison to the Keyence and OCT, especially for larger sample sizes, the same scans can also be used to evaluate internal defects as mentioned in the adaptability considerations. Despite this, compared to the brief time needed for OCT and Keyence measurements, the XRCT received the lowest score.

Based on the results of the Pugh Matrix, the Keyence received the highest score given the criteria weighting. The OCT is close in score but lower due to its limited adaptability, and the XRCT received the lowest score due to ease of use and time to complete. However, it does not directly signify that the Keyence is the ideal method, as it still has limitations regarding safety and requires consideration of tablet potency.

When comparing existing methods, such as SEM, which was not explicitly evaluated in this study, this technique commonly used for particle characterization yields high-resolution images that show the delineation between film coat and core tablet. However, SEM typically requires physical destruction of the tablet, similar to the Keyence method [8]. A further study incorporating SEM may be beneficial to evaluate its efficacy relative to the Keyence, OCT, and XRCT. The current standard method of assessing film coat thickness by percent weight gain is a quick, simple macroscopic technique that requires only an analytical balance and basic calculations. However, it provides only an average thickness for a batch sample and does not offer information regarding coating uniformity or quality. Therefore, it is advisable to use one of the imaging methods evaluated in this study to validate weight gain results and obtain more detailed insights into the tablet and coating characteristics [6].

## 5. Conclusions

This study systematically evaluated the efficacy of OCT and XRCT for quantifying tablet film coat thickness, using measurements obtained via the Keyence Microscope as the reference standard. While there is continuous motivation within Process Analytical Technology to find innovative solutions in pharmaceutical manufacturing, research has predominately focused on in-line large-scale analytical methods such as NIR, Raman Spectroscopy, and Terahertz Imaging. In contrast, there remains a significant knowledge gap regarding robust, lab-scale analytical techniques suitable for drug product development. The aim of this investigation was to broaden the understanding of analytical modalities for precise film coat thickness measurement at the lab scale. Four distinct tablet types, varying in size, coating thickness, and formulation, were employed to assess the performance, accuracy, and limitations of each method.

The Keyence and OCT methods were found to provide the most accurate and precise measurements, especially with the support of the follow-up OCT study, whereas the XRCT also produced accurate results but with precision limited by the equipment’s resolutions capabilities. When evaluating additional criteria including safety, ease of use, adaptability, and time to complete, the Keyence approach was determined to be the most effective method, particularly when used for non-potent products. Although the aim of the study served to primarily evaluate the effectiveness of OCT and XRCT for measuring tablet film coat thickness, the reference method is also a viable technique that cannot be excluded from consideration. The OCT approach is also an efficient method, but it is limited by its capacity to only measure clear-coated tablets. The XRCT is capable of measuring tablets of varying sizes and coatings but requires parameter optimization and has the greatest time requirement, which means it can prove inefficient in a development lab. With this, all three methods are sufficient for measuring film coat thickness, but the optimal method is dependent on individual tablet characteristics. When choosing a method, specifically in the case of purchasing a new instrument, the product pipeline must also be considered. It can be concluded that the Keyence approach is ideal for all non-potent products, the OCT approach for all clear-coated tablets, and the XRCT approach for small tablets and when additional information regarding internal defects is also desired.

## Figures and Tables

**Table 1:** Tablet characteristics.

Tablet	Shape	Approximate Dimensions	Coating Solution
A	Oval	Thickness = 6 mm Width = 9 mm Length = 18 mm	HPMC Triacetin Water
B	Round Bi-Convex	Thickness = 6 mm Diameter = 9 mm	Cellulose Acetate PEG Acetone/Water
C	Standard Convex Mini Tablet	Thickness = 2 mm Diameter = 2 mm	HPMC Triacetin Water
D	Standard Convex Mini Tablet	Thickness = 4 mm Diameter = 3 mm	PLGA Colloidal Silica Acetone

**Table 2:** Sample sizes of tablets for each analytical method.

Tablet	Keyence	OCT	XRCT
A	3	6	3
B	6	6	1 *
C	6	6	6
D	12	12	12

* Limited sample size due to equipment availability at time of testing.

**Table 3:** Refractive index of each coating solution.

Tablet	Refractive Index	RSD
A	1.35	-
B	1.44	2.7%
C	1.35	-
D	1.38	1.6%

**Table 4:** Keyence film coat thickness measurement data.

Tablet	Mean (µm)	RSD	Median (µm)
A	40.9	21.5	39.9
B	139.2	9.2	142.0
C	24.9	25.7	24.5
D	29	15.8	28.8

**Table 5:** Optical coherence tomography film coat thickness measurement data.

Tablet	Mean (µm)	RSD	Median (µm)
A	61.3	15.3	62.6
B	135.9	6.0	134.3
C	33.6	14.9	33.0
D	46	9.8	45.3

**Table 6:** X-ray Computed Tomography film coat thickness measurement data.

Tablet	Mean (µm)	RSD	Median (µm)
A	52.0	27.1	48.0
B	142.1	12.3	136.0
C	23.2	23.7	24
D	30.1	16.9	28.0

**Table 7:** Paired *T*-test *p*-values obtained from tablets B and D comparing Keyence & OCT Data and Keyence & XRCT Data.

Tablet	Keyence & OCT	Keyence & XRCT
B	0.207	0.525
D	0	0.135

## Data Availability

The raw data supporting the conclusions of this article will be made available by the authors on request.

## Abbreviations

The following abbreviations are used in this manuscript:

OCT	Optical Coherence Tomography
XRCT	X-ray Computed Tomography
API	Active Pharmaceutical Ingredient
NIR	Near-Infrared Spectroscopy
SEM	Scanning Electron Microscopy
OEB	Occupational Exposure Band

## Appendix A. Optical Coherence Tomography

**Table A1:** Wasatch Spark OCT parameters.

Setting	Value
numascans	1024
numavgframes	2
imgprocwinminval	0.664835155010223
imgprocwinmaxval	1
dispinitguess	40
dispbcoeff	−40
dispccoeff	19.3846130371094
dispdcoeff	0
trigdelay	100
mirfreq	15
xrampstop	23,811.455078125
xrampstart	23,285.2783203125
yrampstop	23,718.6328125
yrampstart	19,629.392578125
focus	121
continuousupdate	True
foci	158
pathlengthvalue	960
polarityquartervalue	0
polarityhalfvalue	0
opacitywindow	40,000
Opacitylevel	15,000
Windowwidth	1
Windowlevel	0.5
use3dblackbackground	True
use3dtrackball	True
opacitymax	0.9

## Appendix B. XRCT Parameters

**Table A2:** Tablet A XRCT parameters.

Setting	Value
Height	19 mm
Source	80 kV × 125 uA
Exposure	48 ms
Rotation Step	0.1
Frame Averaging	10
Resolution	7 µm/pixel
Aluminum Filter	Yes

**Table A3:** Tablet B XRCT parameters.

Setting	Value
Height	32 mm
Source	60 kV × 166 uA
Exposure	80 ms
Rotation Step	0.4
Frame Averaging	5
Resolution	7 µm/pixel
Aluminum Filter	Yes

**Table A4:** Tablet C XRCT parameters.

Setting	Value
Height	16.5 mm
Source	30 kV × 212 uA
Exposure	110 ms
Rotation Step	0.1
Frame Averaging	10
Resolution	4 µm/pixel
Aluminum Filter	No

**Table A5:** Tablet D XRCT Parameters.

Setting	Value
Height	17 mm
Source	30 kV × 212 uA
Exposure	110 ms
Rotation Step	0.1
Frame Averaging	10
Resolution	4 µm/pixel
Aluminum Filter	No
